# A simple combined approach using anterior transpetrosal and retrosigmoid approach: A case report

**DOI:** 10.3389/fsurg.2023.1094387

**Published:** 2023-02-27

**Authors:** Ryota Tamura, Ryo Ueda, Kosuke Karatsu, Taichi Sayanagi, Kento Takahara, Utaro Hino, Takashi Iwama, Hirotsugu Nogawa, Masato Nakaya, Takashi Horiguchi, Masahiro Toda

**Affiliations:** Department of Neurosurgery, Keio University School of Medicine, Tokyo, Japan

**Keywords:** anterior transpetrosal approach, retrosigmoid approach, lateral suboccipital approach, combined transpetrosal approach, posterior cranial fossa, skull base

## Abstract

**Background:**

A combined transpetrosal approach (CTP) is often used for large lesions in the posterior cranial fossa (PCF). Although CTP provides a wide surgical corridor, it has complex and time-consuming bony work of mastoidectomy and cosmetic issues. Here, we describe a simple combined surgical technique to approach the supratentorial region, anterolateral surface of the brainstem, petroclival region, and foramen magnum by drilling only the petrous apex with a combination of retrosigmoid approach (RA).

**Clinical presentation:**

A 27-year-old female was referred with extra-axial left cerebellopontine angle space-occupying epidermoid cyst extending to the prepontine cistern, anterior to the basilar artery, superior to the chiasma, and caudally to the foramen magnum. A one-stage surgical procedure using the anterior transpetrosal approach (ATP) and RA was performed after one-piece temporal-suboccipital craniotomy. These two approaches complemented each other well. Near-total removal was achieved.

**Conclusion:**

A one-stage surgical procedure using ATP and RA provides the wider viewing and better visualization of the PCF with minimal technical difficulty.

## Introduction

The anterior transpetrosal approach (ATP) is a skull base surgical procedure that can provide an operative view of the petroclival and supratentorial region (1). However, this surgical approach is not appropriate for lesions located around the lower cranial nerves (LCN) and the hypoglossal nerve (2). Posterior transpetrosal approaches, including the retrolabyrinthine, translabyrinthine, and transcochlear approaches, are based on the standard mastoidectomy, which is mainly used for large lesions in the posterior cranial fossa (PCF) from the top of the clivus to the jugular foramen (3). Furthermore, wide surgical exposure of the cerebellopontine angle (CPA) and petroclival region can be obtained *via* the combinational approach of anterior and posterior transpetrosal procedures (4). A combined transpetrosal approach (CTP) can minimize cerebellar and temporal lobe retraction (5). However, it may have complex and time-consuming bony work of mastoidectomy and cosmetic issues (5).

The retrosigmoid approach (RA) is indicated for resection of infratentorial tumors (6). RA is simple and quick, and it does not need temporal lobe retraction. The disadvantage is the limited access to the prepontine and supratentorial regions (6). Anterior petrosectomy can overcome the limitations associated with RA (7). Therefore, the combination of ATP and RA has the possibility to result in many options for obtaining wide surgical exposure of the PCF and overcome the complex procedure of mastoidectomy.

In this report, we describe a simple one-stage operation to approach the anterolateral surface of the brainstem, petroclival region, and foramen magnum (FM) by drilling only the petrous apex with a combination of RA.

## Case presentation

### Patient characteristics

**Figure 1:**
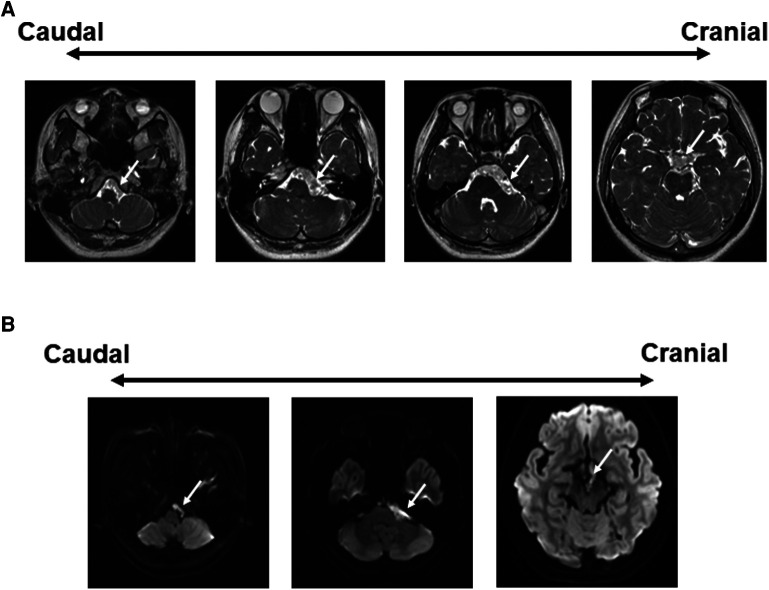
Preoperative radiographic findings. (**A**) FIESTA demonstrates an extra-axial lobulated mass at the left CPA, extending to the prepontine/interpeduncular cisterns as well as the ipsilateral FM, compressing the brainstem, and cranial nerves. There is also an extension into the ipsilateral IAC with encasement of the basilar artery. White arrow: tumor. (**B**) Axial DWI image shows bright signals in the CPA cistern. CPA, cerebellopontine angle; FM, foramen magnum; IAC, internal auditory canal.

A 27-year-old female presented with a 4-month history of left trigeminal neuralgia and left hearing impairment followed by dizziness and headache. Fast imaging employing steady-state acquisition (FIESTA) showed an extra-axial left CPA space-occupying lesion extending to the prepontine cistern, anterior to the basilar artery, superior to the chiasma, and caudally to the FM (Figure 1A). Tumor extended through the left internal auditory canal (IAC). It has a lobulated outline with low T1-weighted imaging (T1WI), high T2WI, and heterogeneous fluid-attenuated inversion recovery (FLAIR) signals with evident diffusion restriction (Figure 1B). No post-contrast enhancement was seen. Preoperative axial computed tomography (CT) scan showed the development of air cells in the petrous apex. The peritubal air cell tract connecting around the Eustachian tube from the apical area of the petrous apex was identified (8). A one-stage surgical procedure using ATP and RA was planned.

### Skin incision and craniotomy

**Figure 2:**
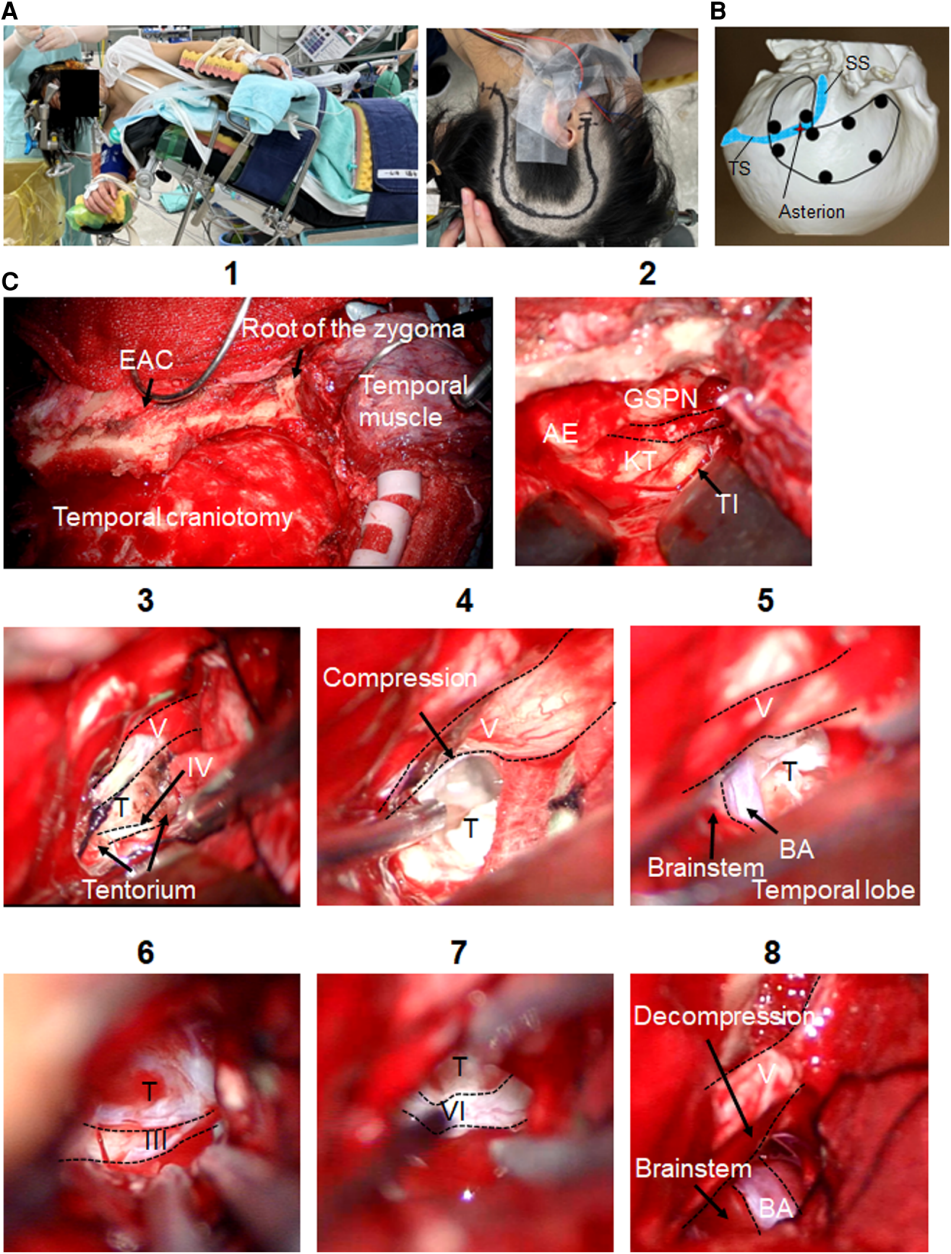
Intraoperative findings of the ATP. Microsurgical maneuvers of the ATP are shown. (**A**) Skin incision and patient's position (Park bench) are shown. (**B**) Burr-hole placement for the one-piece temporal-suboccipital craniotomy is shown. (**C**) Surgical field of the ATP is shown (1). The MCF dura is elevated toward the petrous ridge. The middle meningeal artery is coagulated and divided. After the GSPN is preserved, the landmarks for drilling Kawase's quadrangle are identified (2). The tentorium is opened exposing the trigeminal nerve superior to petroclival tumor. Trochlear nerve is exposed coursing over tumor before entering tentorium (3). Trigeminal nerve is compressed by tumor (4). Tumor removal of the prepontine cistern and supratentorial region is done. BA, brainstem (5), and oculomotor nerve (6) are identified. The lower limit of this exposure is the abducens nerve (7). Trigeminal nerve decompression is done (8). ATP, anterior transpetrosal approach; MCF, middle cranial fossa; AE, arcuate eminence; BA, basilar artery; EAC, external auditory canal; GSPN, greater superficial petrosal nerve; KT, Kawase's triangle; T, tumor; TI, trigeminal impression; TS, transverse sinus; SS, sigmoid sinus.

The head was placed in the horizontal position, with the body in the lateral position (Figure 2A). The skin incision began 1 cm anterior to the tragus, went cranially until the superior temporal line, turned posteriorly, then turned caudally approximately 6 cm posterior to the external auditory canal, and ended around the FM (Figure 2A). The skin flap was elevated off the temporal muscle, pericranium, and suboccipital muscles. A temporalis fascia flap was harvested to aid subsequent dural closure. The temporal muscle was elevated and retracted anteriorly. The suboccipital muscles were elevated and retracted laterally.

A neuronavigation system was used for outlining the transverse and sigmoid sinuses. Multiple burr holes were placed as described in Figure 2B. Once the one-piece temporal-suboccipital craniotomy was completed, the bone flap was lifted off, exposing part of the supratentorial dura, PCF dura, transverse sinus, and sigmoid sinus. The squamous temporal bone was drilled flat to the middle cranial fossa (MCF) floor. Sigmoid sinus was carefully skeletonized.

### Anterior transpetrosal approach

Microsurgical maneuvers of the ATP are shown in Figure 2C. The MCF dura is elevated toward the petrous ridge. The middle meningeal artery was coagulated and divided. Interdural temporal elevation was performed to preserve the greater superficial petrosal nerve. Dura propria overlying the trigeminal nerve was incised sharply, and dural elevation was carried out progressively. After dural elevation was turned posteriorly toward the arcuate eminence, the petrous ridge was reached. The landmarks for drilling Kawase's quadrangle were identified. Petrous apex was removed to gain access to PCF. The temporal dura matter was opened parallel to superior petrosal sinus (SPS). PCF dura matter was opened below SPS. The SPS was coagulated and cut. The tentorium was opened exposing the trigeminal nerve superior to petroclival tumor component, preserving the trochlear nerve. The porus trigeminus was opened to expose trigeminal ganglion in Meckel's cave. Near-total resection with cyst capsules of prepontine and supratentorial tumor was achieved, preserving oculomotor, trochlear and trigeminal nerves, basilar artery, superior cerebellar artery, and brainstem. The lower limit of this surgical exposure around abducens nerve was exposed. A small amount of tumor remained around the optic chiasm.

### Retrosigmoid approach

**Figure 3:**
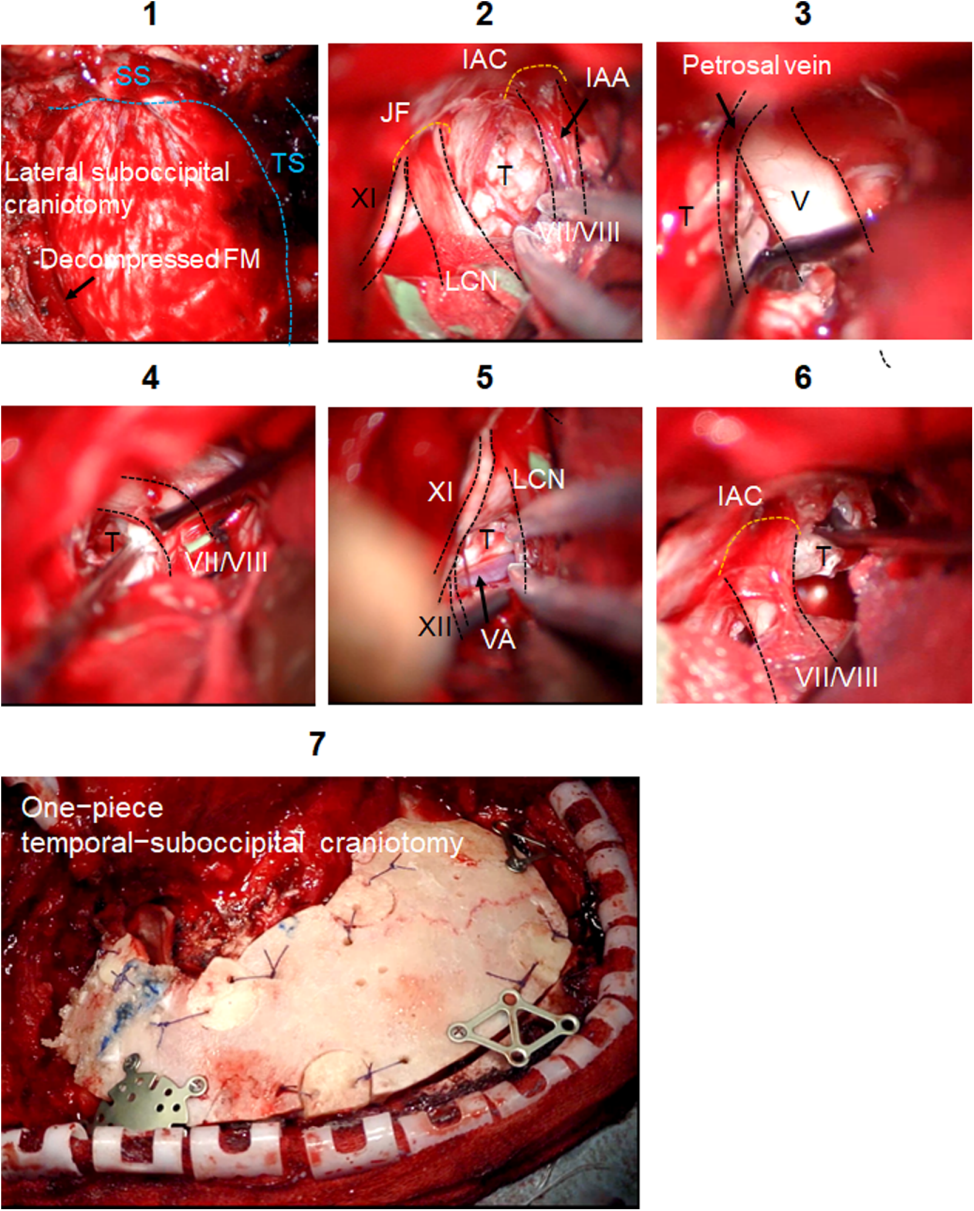
Intraoperative findings of the LSO. Microsurgical maneuvers of the LSO are shown. Surgical field of the LSO is shown. SS is skeletonized. Foramen magnum decompression is performed (1). The dura is incised in a C-shape fashion, and the cerebellar hemisphere is gently retracted to expose the cerebellomedullary cistern (2). Tumor removal around the trigeminal nerve (3), facial/vestibulocochlear nerves (4), and LCN (5) is done. Tumors growing below vertebral artery (VA) and hypoglossal nerve are removed (5). Tumor extending into the IAC is gently removed with curette (6). The decompression is achieved by removing bone posterior and inferior to the IAC. The one-piece temporal-suboccipital bone flap is replaced and attached to the surrounding bone with titanium plates and screws (7). IAC, internal auditory canal; FM, foramen magnum; IAA, internal auditory artery; JF, jugular foramen; LCN, lower cranial nerves; T, tumor; TS, transverse sinus; SS, sigmoid sinus.

Microsurgical maneuvers of the RA are shown in Figure 3. FM decompression was performed to increase the volume of the PCF. The dura was incised along the inferior border of the transverse sinus, and then it was carried inferiorly posterior to the sigmoid sinus. The operative corridor was widened by retracting the petrosal surface of the cerebellum. A smooth dissection was applied to detach the capsule of epidermoid cyst from facial/vestibulocochlear nerves, LCN, and hypoglossal nerve. Complete resection with cyst capsules of CPA and foramen magnum was achieved. After the IAC decompression, tumor component in the IAC was removed.

This surgery took 10 h 33 min in total (ATP: 3 h 15 min; RA: 4 h 25 min; other general procedures: 2 h 53 min).

### Postoperative course

**Figure 4:**
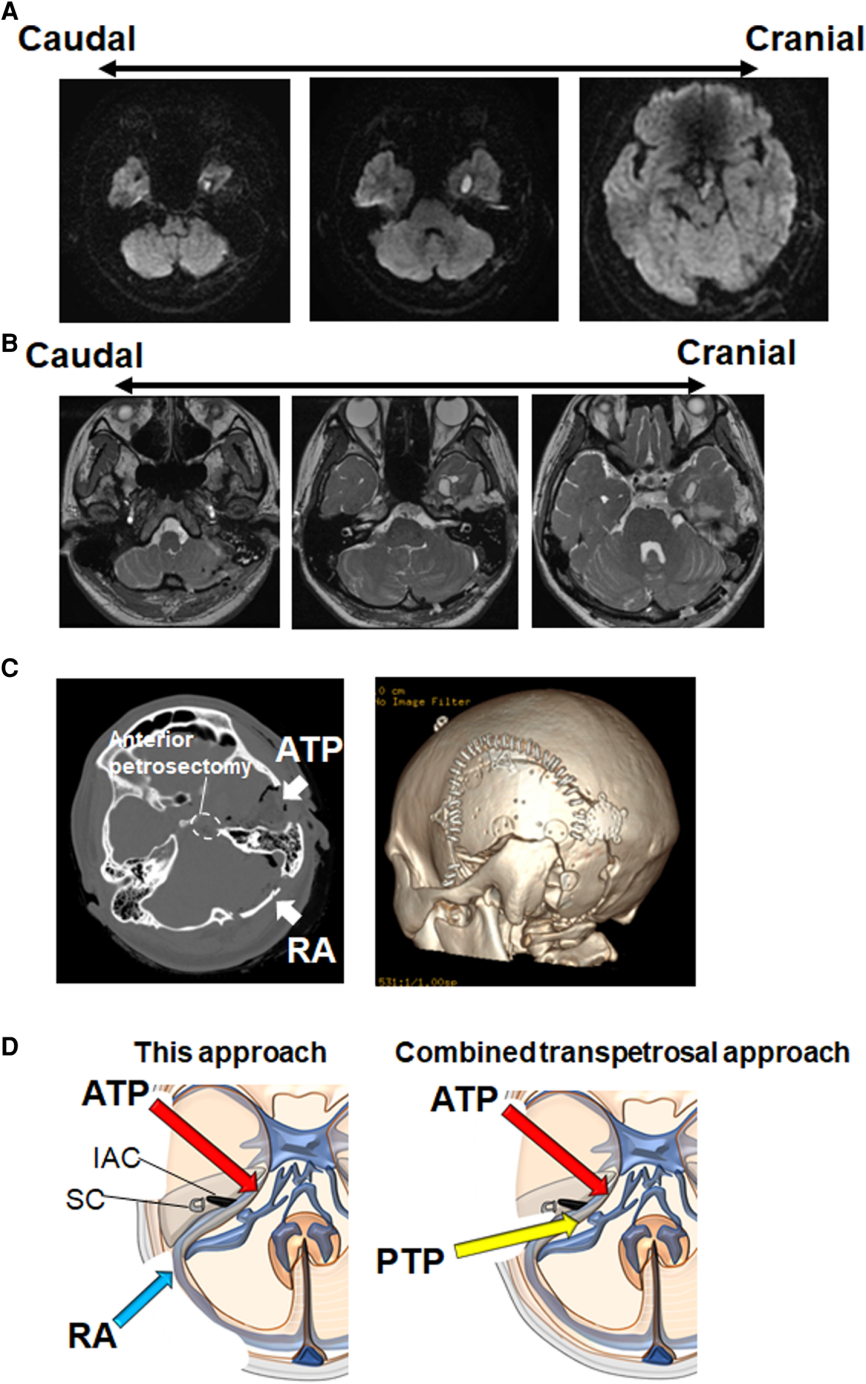
Postoperative radiographic findings. (**A**) Postoperative DWI shows near-total removal of epidermoid cyst in the supra/infra tentorial regions. A small amount of tumor remains around the optic chiasm. (**B**) Postoperative FIESTA shows that the decompression of trochlear nerve, trigeminal nerve, facial/vestibulocochlear nerves, LCN, and hypoglossal nerve is achieved. (**C**) Head CT bone window images show the one-piece temporal-suboccipital craniotomy (left panel: axial view, right panel: three-dimensional reconstruction). (**D**) Schematic diagram illustrating the difference between this approach and CTP. FIESTA, fast imaging employing steady-state acquisition; LCN, lower cranial nerves; CTP, combined transpetrosal approach; ATP, anterior transpetrosal approach; IAC, internal auditory canal; PTP, posterior transpetrosal approach; RA, retrosigmoid approach; SC, semicircular canal.

Postoperative Diffusion-weighted imaging (DWI) displayed near-total tumor removal (Figure 4A). FIESTA showed that the decompression of all cranial nerves is achieved in the PCF (Figure 4B). Craniotomy size is shown in Figure 4C. Trigeminal neuralgia, headache, and dizziness were completely reduced. Abducens nerve palsy has occurred following the operation, which completely improved 1 month after the operation. The patient was discharged 10 days after the operation.

## Discussion

In general, CTP is used for large lesions in the PCF. Although some authors simplified CTP by minimizing the petrosectomy and mastoidectomy (9, 10), most neurosurgeons may not be familiar with these complex procedures. The RA, with which most neurosurgeons are familiar, is versatile for the treatment of different pathologies in PCF (6). This procedure facilitates the rapid identification of the neurovascular structures in CPA. However, RA could not provide an adequate exposure to the clivus and anterior/superior part of the brainstem (6). Because anterior petrosectomy can overcome the limitations associated with RA, a two-stage approach with ATP and RA has been already performed (11). In this report, a one-stage surgical procedure using ATP and RA was proposed. This approach may be simple and quick for most neurosurgeons compared with CTP. Because mastoidectomy is not required in this approach, we may avoid sigmoid sinus-associated complications. The inferior portions of the CPA are much more clearly observed *via* this approach than *via* the CTP (Figure 4D). Furthermore, for hypervascular tumors such as meningioma, we recommend the RA procedure after the ATP, because the early coagulation of feeding arteries (the tentorial artery and the middle meningeal artery) can be performed during the ATP.

However, there may be disadvantages with this approach. During the present simultaneous procedures, the microscope's visual axis has to be tilted considerably. The operator needs to change the standing position during the operation. With conventional equipment, this procedure may force the surgeon into an uncomfortable position for the manipulation of RA. The best view can be obtained through bed rotation in both directions and will mitigate the surgeon's fatigue at the same time in this combined approach. An exoscope permits us to perform stable microsurgery in a comfortable posture regardless of the angle of the operative visual axis (12). In addition to the potential hearing loss, other RA-related morbidities include cerebellar edema from retraction and venous congestion from venous thrombosis or sacrifice. Intraoperative use of an endoscope may extend the view to the corner looking at the perforator that could not be provided by microscopic view, preventing the complications (13).

Further studies are warranted to confirm the merits of this combined strategy. This is a case report and thus should be further assessed using clinical studies to evaluate the practical advantages and disadvantages of this approach.

## Conclusions

A one-stage surgical procedure using ATP and RA provides wider viewing and better visualization of the PCF with minimal technical difficulty. This simple combined procedure may be versatile for the treatment of PCF pathology.

## Data Availability

The original contributions presented in the study are included in the article/Supplementary Material, further inquiries can be directed to the corresponding author.
